# Towards sustainable mental health promotion: trial-based health-economic evaluation of a positive psychology intervention versus usual care

**DOI:** 10.1186/s12888-018-1825-5

**Published:** 2018-08-23

**Authors:** Marijke Schotanus-Dijkstra, Constance H. C. Drossaert, Marcel E. Pieterse, Jan A. Walburg, Ernst T. Bohlmeijer, Filip Smit

**Affiliations:** 1 Centre for eHealth and Well-being Research, Department of Psychology, Health and Technology, University of Twente, P.O. Box 217, 7500 AE Enschede, The Netherlands; 2 Department of Public Mental Health, Trimbos Institute, Utrecht, The Netherlands; 3 Department of Epidemiology and Biostatistics, EMGO Institute for Health and Care Research, VU University Medical Centre, Amsterdam, The Netherlands; 4 Department of Clinical, Neuro and Developmental Psychology, EMGO Institute for Health and Care Research, VU University, Amsterdam, The Netherlands

**Keywords:** Cost-effectiveness, Mental well-being, Guided self-help, Prevention, Positive psychology, Randomized controlled trial

## Abstract

**Background:**

Mental well-being could be promoted and protected by positive psychology (PP) based interventions. Such interventions may be appealing for people at risk of anxiety and depressive disorders, but health-economic evaluations are scarce. The aim was to examine the cost-effectiveness of a PP intervention.

**Methods:**

Participants with suboptimal levels of mental well-being were randomly assigned to an email guided PP-intervention (*n* = 137) or a wait-list control group (*n* = 138) with access to usual care (UC). At baseline and 6 months follow-up, data were collected on health care costs. Outcomes of interest were flourishing mental health and treatment response on anxiety and depressive symptoms.

**Results:**

Bootstrapped mean incremental cost-effectiveness ratios were €2359 ($2899) for flourishing, €2959 ($3637) for anxiety and €2578 ($3168) for depression, suggesting appreciable health gains for low additional costs. At a willingness to pay ceiling of €10,000 ($12,290) for a treatment response, the probability that the intervention is deemed cost-effective ranged between 90 and 93%.

**Conclusions:**

The guided PP intervention appears to be a promising strategy as seen from both a public health and a health-economic perspective, especially when there is some willingness to pay. When the PP-intervention is scaled up, then outcome monitoring is recommended to better guarantee the longer term cost-effectiveness of the intervention.

**Trial registration:**

The Netherlands National Trial Register NTR4297. Registered on 29 November 2013. The NTR is part of the WHO Primary Registries.

## Background

A new cost-effective strategy for the prevention of anxiety and depressive disorders might be to promote a flourishing mental health state in people with low or moderate levels of mental well-being [1, 2]. Earlier studies demonstrated that these people have an increased health care use^3^ with concomitant health care costs and productivity losses [3–5]. Flourishing is defined as the presence of *high levels* of emotional well-being (e.g. life-satisfaction, positive affect) in combination with *high levels* of social and psychological well-being (e.g. social contribution, positive relationships, self-acceptance, purpose in life) [6, 7].

Longitudinal studies have shown that flourishing helps to protect against first-onset and recurrence of diagnosed mood and anxiety disorders [8, 9]. Other promising results stem from two of our randomized controlled trials demonstrating that individuals are able to improve their well-being up to flourishing levels using email guided bibliotherapy based on a positive psychology or related framework [10, 11]. The same studies also found beneficial effects on anxiety and depressive symptoms, suggesting that deteriorating mental health could be prevented by such self-help interventions. Self-help books are widely available and have the purpose to reach the lay public [12]. However, it is yet unknown whether positive psychology (PP) based bibliotherapy programs are cost-effective [13].

To our knowledge, only one prior study evaluated the cost-effectiveness of a positive psychology intervention, which was an online multicomponent intervention for people with mild to moderate depressive symptoms [14]. This non-guided web-based intervention contained psycho-education and practical exercises about goal setting, positive emotions, positive relations, mindfulness, optimism and mastery. Participants in the experimental condition could independently select modules and exercises to tailor the intervention to their needs. At six months follow-up, the intervention was not effective in improving the primary outcome of mental well-being, but it was effective in reducing depressive symptoms compared to a wait-list (usual care) control group. However, the online intervention was not found to be cost-effective from a societal cost-effective perspective on any outcome measure [14]. According to the authors, these somewhat disappointing findings might be partly attributed to the lack of compliance, since only 10% of the participants completed at least one module as recommended [14, 15]. Therefore, a more cost-effective self-help format for positive interventions might include some therapist involvement to increase adherence. The present economic evaluation uses our trial data of an early bibliotherapy intervention for people with suboptimal levels of mental well-being [11]. We hypothesized that this PP-based self-help intervention with some guidance over the Internet was also cost-effective relative to usual care alone.

## Methods

### Design and participants

The non-blinded randomized controlled trial was conducted in two parallel groups, with computerized randomization using Excel (1:1 allocation) and stratified for gender and education (low, medium, high) performed by the first author. For each group, a contact list was created in Qualtrics, making it possible to send personal emails to participants (e.g. including the result of the randomized assignment) without interference of the researcher. A sample size of 132 participants per condition was required to provide a statistical power of 80%, two-sided with α of 5% and compensating for a 25% drop-out rate, to detect a small to medium effect size (Cohen’s *d* of 0.40) on the main outcome i.e. mental well-being (a continuous measure) [16, 17]. Although the primary outcome of the current study was the binary measure of mental well-being, a similar sample size was needed when the power calculation was adjusted to a χ^2^ test and intention-to-treat data (*n* = 126 per group). Participants aged 18 years or older were recruited from the general Dutch population in January 2014 via national advertisements calling for people who were motivated to actively work on their “well-being and resilience”. The participants were willing to invest an average time of 4 h per week for 9 weeks and had access to email and the Internet. Interested participants completed a contact form via a research website, and received an online informed consent procedure per email before they could access the online screening survey. Eligible participants were excluded when they were younger than 18 years of age, already possessed a flourishing mental health status as measured with the Mental Health Continuum-Short Form (MHC-SF); scoring 4 or 5 on at least one emotional well-being item together with a score of 4 or 5 on at least 6 of the 11 social and psychological well-being items [7, 18] or when they presented scores above 10 on at least one subscale of the Hospital Anxiety and Depression Scale (HADS), indicating moderate to severe anxiety or depressive symptoms [19]. Also, participants had to complete the screening and baseline questionnaire because randomization took place after baseline.

In total, 518 participants were interested in participating in the study, of which 243 participants had to be excluded [11]. The final sample of 275 participants were allocated to the intervention group (*n* = 137) or the wait-list control group (*n* = 138). The trial protocol was approved by the Ethics Committee of the University of Twente (no. 13212) and registered at The Netherlands Trial Register (NTR4297). The design of the study [20] and its main findings [11] are published elsewhere.

### Interventions

Participants in the intervention group received (1) the self-help book *This is Your Life* [21], (2) a 9-week time schedule for reading the book with recommended exercises, and (3) weekly email support from a personal counselor. The book consists of eight chapters containing psycho-education, theoretical background information, and a variety of evidence-based exercises from positive psychology, but also from mindfulness and acceptance and commitment therapy. The purpose of the book is to improve an individual’s capacity to savor positive emotions, discover and use character strengths, encourage flow and an optimistic attributional style, develop self-acceptance and compassion, learn to cope with adversity (resilience), and encourage to share and connect with others [20]. The chapter about discovering and using character strengths was spread out over 2 weeks in the time schedule. Participants had 8 to 12 weeks to complete the program. A full description of each chapter and recommended exercises can be found elsewhere [20].

Once a week, participants emailed their personal counselor with their experiences about the chapter and corresponding exercises. Five positive psychology students each guided 25 participants and the first author guided the remaining participants. The counselors were trained in providing email support during a study course plus a one-day workshop. In addition, they attended weekly supervision meetings. The email correspondence was aimed at increasing adherence. On average, participants reported that they had completed 6.4 (out of the 8) chapters (SD = 2.4) and had sent 6.4 extensive emails (SD = 3.62), indicating adequate adherence to the protocol [11].

Participants in the wait-list control group received the self-help book *This is Your Life* and the 9-week time schedule after completing the 6 months assessment. Participants in both conditions had unrestricted access to usual care.

### Health related outcomes

The primary outcome was flourishing mental health and the secondary outcomes were anxiety and depressive symptoms. Self-reported data were obtained from online questionnaires at baseline and 6 months follow-up.

### Flourishing

The 14-item MHC-SF measures mental well-being on a continuous scale but can also classify people into (1) flourishing mental health, (2) moderate mental health or (3) languishing mental health [18, 22], although we put the latter two categories together because there were few people with languishing mental health in the current sample at baseline (4.4%). In a cost-effectiveness study, hard currency (measured at the interval level) cannot be meaningfully related to health benefits that are measured at the (“elastic”) ordinal measurement scale. It is economically more meaningful to relate hard currency to a binary outcome such as treatment response, where treatment response is clearly defined as reliable change [23] or as a transition from one health state (e.g. languishing or moderate mental health) to another (e.g. flourishing mental health).

The first three items of the MHC-SF measure emotional well-being (i.e. happiness, interest, life-satisfaction), the next five items measure social well-being (i.e. social contribution, social integration, social actualization, social acceptance, social coherence) and the last six items tap into psychological well-being (i.e. self-acceptance, mastery, positive relations, personal growth, autonomy, purpose in life). Each item was scored on a 6-point scale from 0 (never) to 5 (almost always). Flourishing is theoretically operationalized as scoring 4 or 5 on at least one emotional well-being item together with a score of 4 or 5 on at least 6 of the 11 remaining items, which parallels the DSM-IV approach to diagnose a major depression [7, 18, 24]. The Dutch version of the MHC-SF has shown good psychometric properties [22] and showed good internal consistency in the current study (α = 0.88).

### Anxiety and depressive symptoms

Anxiety and depressive symptoms were measured with the HADS-A and HADS-D respectively. Each subscale has 7 items with scores ranging from 0 to 3. Total summed scores range from 0 to 21, with higher scores indicating greater anxiety or depressive symptom severity. The HADS has shown good psychometric properties in the Dutch population [25, 26] and showed good internal consistency in the current sample (α = 0.76 for both subscales). To measure treatment response on these scales, Jacobson and Truax’ method [23] was applied to obtain the reliable change index to distinguish between treatment responders and non-responders. The reliable change index was calculated as *x*_2_ – *x*_1_ / S_diff_, where *x*_2_ is the post-test score and *x*_1_ is the baseline score. S_diff_ is the standard error of difference between the pre- and post-test scores which is calculated as √(2(S_E_)^2^), where the standard error of measurement (S_E_) is calculated as SD√(1 – *r*_*xx*_). *r*_*xx*_ is the test-retest reliability of the measure, which was 0.89 for the HADS-A and 0.86 for the HADS-D [26]. A treatment responder was estimated to be a pre-post change of at least 2.22 points on the anxiety subscale and 2.55 points on the depression subscale, taking the 95% criterion into account (*z* = 1.96).

### Resource use and costing

The current study adopts a health sector perspective in accordance with national UK and US guidelines [27, 28]. Therefore, direct medical costs (health service use), direct non-medical costs (travel costs to health services) and intervention costs were included and not productivity losses. Resource use was measured with the Medical Consumption Questionnaire (MCQ) for three periods: 3 months before baseline (T0), baseline to 3 months follow-up (T1) and 3 to 6 months follow-up (T2) [29]. All costs are expressed in euros (€) for the reference year 2014. The main results are also expressed in US dollars ($). The general purchasing power parity (PPP) was used for conversion of the € to $ for 2014 (US$1.00 = NL€0.814) [30].

### Direct medical and non-medical costs

Table 1 displays an overview of health service units (contacts or hours) and their standard unit cost price as reported in the Dutch guidelines for health-economic evaluations [31]. Health service costs per participant were calculated by multiplying the utilized health service units of each participant in the past 3 months with the standard unit cost price of that service. Travel costs for each health service visit were calculated by multiplying the average distance to that service according to the Dutch guidelines (see Table 1) with the costs per km (€0.19). Parking costs were added to the travel costs which amounted to €3 per visit. Other costs of participants or its family members outside formal health care (e.g. informal care) which might have had a direct relation with an illness were not included in this health-economic evaluation because no severe (chronic) disorders were investigated and the sum of these costs would be limited.

**Table 1:** Unit cost price for direct medical and direct non-medical costs by the reference year 2014

	Direct medical costs		Direct non-medical costs	
Health service type	Unit	Unit cost price	km	Unit cost price
Family doctor – standard consult	Contact	33	1,1	3.21
Family doctor – mental health	Contact	66	1,1	3.21
Family doctor – home visit	Contact	50	NA	NA
Company doctor	Contact	33	17,6	6.34
Social worker	Contact	65	5	3.95
Regional mental health center	Contact	112	10	4.90
Regional addiction center	Contact	112	10	4.90
Independent psychologist, psychotherapist, psychiatrist	Contact	94	7	4.33
Psychologist, psychotherapist, psychiatrist in hospital^a^	Contact	91	7	4.33
Self-help group	Hour	14	7	4.33
Alternative healer^b^	Contact	55	5	3.95

^a^Unit cost price was based on a weighted mean of a general and academic hospital

^b^Unit cost price was based on own calculation as weighted average of homeopath and acupuncturist

### Intervention costs

Each participant in the intervention group received the self-help book *This is Your Life* which was valued at €25 in 2014 as found in a large and representative online bookstore in The Netherlands. Each participant also received personal email support. Although the current study used students as counselors, in real-life health care settings these will be replaced by the family doctor’s mental health nurse at €17 per consult [31]. We assume an average of nine email contacts, which puts the costs at €17*9 contacts = €153 per participant. Additional costs were incurred for recruitment, screening and training costs for the mental health nurse at €44 per participant in a real-world setting. In total, the estimated intervention costs were €222 ($273) per participant.

### Analysis

#### Statistical analyses

Analyses of the health related outcomes were carried out in accordance with the intention-to-treat principle. Missing data on the MHC-SF, HADS-A and HADS-D at T2 were imputed using the expectation maximization (EM) algorithm in SPSS (IBM, Chicago, Ill., USA) version 22.0. The results of these health effects were also reported in our prior trial but repeated here for clarity [11].

#### Cost-effectiveness analyses

The Consolidated Health Economic Evaluation Reporting Standards (CHEERS) were followed [27]. Therefore, the intention-to-treat principle was also applied to the cost data and these were imputed using EM. In order to compare our results with other cost-effectiveness studies, costs were annualized by multiplying the costs of three months by 4. No discounting of costs and effects was applied because the study’s follow-up did not exceed one year [27]. Furthermore, the conditions were compared as if operating under steady-state conditions. This means that it is assumed that the health care costs in the past 3 months (as well as the health gains in the past 4 weeks) as measured at the 6 month follow-up are representative for a whole year with the proviso that there were no significant baseline differences between the conditions. Therefore, only the annualized 6 months health care costs (including the intervention costs in the intervention condition) were used for calculating the incremental cost-effectiveness ratio (ICER). The ICER is calculated as (*C*_1_–*C*_0_)/(*E*_1_–*E*_0_), where *C* is the average annualized per-participant health care costs in the experimental and control condition (i.e. incremental costs) and *E* is the proportion of flourishers or treatment responders on the HADS (i.e. incremental effects). The subscripts 1 and 0 refer to the intervention and control condition, respectively. The ICERs represent the incremental costs per additional treatment responder in the intervention as compared to the control group.

A Microsoft Excel macro was used to simulate 2500 ICERs in a non-parametric bootstrap procedure. With this resampling procedure, each estimated ICER was plotted in a cost-effectiveness plane. In this plane, the costs are presented on the x-axis and the health outcomes (i.e. flourishing, anxiety, depression) on the y-axis. When the dots are mainly plotted in the northwest (NW) quadrant (higher costs, less health) or southeast (SE) quadrant (lower costs, health gains), this means that a clear decision can be made from a cost-effective perspective; i.e. the intervention is unacceptable (dominated by usual care) in the NW quadrant and acceptable (dominant) in the SE quadrant compared to the control group. However, the northeast (NE) quadrant (higher costs, health gains) and southwest (SW) quadrant (lower costs, less health) require a more advanced decision-making to balance higher or lower costs against greater or lesser health gains, for which a cost-effectiveness acceptability curve is being used. This curve provides insight into the probability of accepting an intervention relative to a control condition. In the present study, this curve displays hypothetical willingness to pay (WTP) ceilings (€0 - €30,000) for gaining one additional treatment responder on flourishing, anxiety and depressive symptoms respectively (on the x-axis) and graphs the likelihood that the PP intervention is deemed to be of acceptable cost-effectiveness (on the y-axis).

### Sensitivity analysis

The analyses were repeated for three different scenarios to examine the robustness of the results. In scenario A, the intervention costs were based on the actual number of emails sent by the personal counselor to the participant (not the assumed maximum of 9 emails) and then multiplied by the costs for consulting a family’s doctor mental health nurse (€17). In scenario B, the intervention costs were raised by including the time investment of participants (valued at 3 h*9 weeks*€14) amounted to a total of €600 per participant. In scenario C, completers-only analyses were performed, using data of the 112 participants in the intervention condition and 125 participants in the control condition who completed all measurements at T2.

## Results

### Sample characteristics

Participants were predominantly female (85.8%), higher educated (74.5%) and in paid employment (68.4%). Mean age was 47.8 years (SD = 10.9). The mean score of the total sample for mental well-being was 2.57 (SD = 0.63), for anxiety symptoms 7.28 (SD = 2.41) and for depressive symptoms 5.80 (SD = 2.47). There were no significant between-group differences regarding participant characteristics, main outcome measures and resource costs.

### Health effects

At 6 months, there were 42 participants (30.7%) with flourishing mental health in the intervention condition and 16 participants (11.6%) in the control condition (χ^2^ = 15.01, *df* = 1, *P* = < 0.001), as has also been reported previously [11]. The incremental effect (i.e. the proportion of flourishers in the experimental group minus the proportion of flourishers in the control group) was 0.31–0.12 = 0.19. Similar effects were found for anxiety and depressive symptoms. For the HADS-A, 57 participants (41.6%) in the intervention group met the criteria for treatment response compared to 27 participants (19.6%) in the control group (χ^2^ = 15.74, *df* = 1, *P* = < 0.001). For the HADS-D, these number of participants were 59 (43.1%) in the intervention group and 30 (21.7%) in the control group (χ^2^ = 14.29, *df* = 1, *P* = < 0.001). The incremental effects were 0.42–0.20 = 0.22 for anxiety symptoms and 0.43–0.22 = 0.21 for depressive symptoms.

### Costs

At baseline, total average annualized direct medical and direct non-medical costs were €581 (SD = €1190) in the intervention condition and €675 (SD = €1246) in the control condition (Table 2). At T1, these costs were €432 (SD = €983) and €658 (SD = €1224) respectively. At T2, these costs were €506 (SD = €1001) for the guided self-help intervention and €488 (SD = €1190) for usual care. These T2 annualized costs were used for the cost-effectiveness analyses, adding the intervention costs of €222 to the intervention condition. Therefore, the annualized incremental costs at the trial’s follow-up were €728–€488 = €240 ($295).

**Table 2:** Per participant annualized costs in Euros (€) by condition, for 3 months prior to baseline, 0–3 months during intervention (t0-t1) and 3–6 months after intervention (t1-t2)

	Baseline Mean (SD)	0–3 months Mean (SD)	3–6 months Mean (SD)
Wait-list control group (*n* = 138)			
Direct medical costs	637.16 (1185.66)	620.49 (1167.22)	462.97 (1137.84)
Direct non-medical costs	38.14 (61.87)	37.50 (59.61)	25.36 (52.71)
Intervention costs	NA	NA	NA
Total costs	675.30 (1245.89)	657.99 (1223.89)	488.33 (1189.92)
Self-help with email support (*n* = 137)			
Direct medical costs	546.83 (1128.45)	408.09 (939.50)	478.05 (956.73)
Direct non-medical costs	34.07 (63.50)	23.58 (44.69)	27.77 (45.94)
Intervention costs	NA	222	NA
Total costs	580.90 (1190.07)	653.66 (982.97)	505.82 (1001.27)^a^

^a^For further analyses, these annualized costs were included plus the intervention costs of €222. The total mean costs amounted to 727.82 (SD = 1001.27) at 6 months

### Cost-effectiveness: Flourishing

**Fig. 1:**
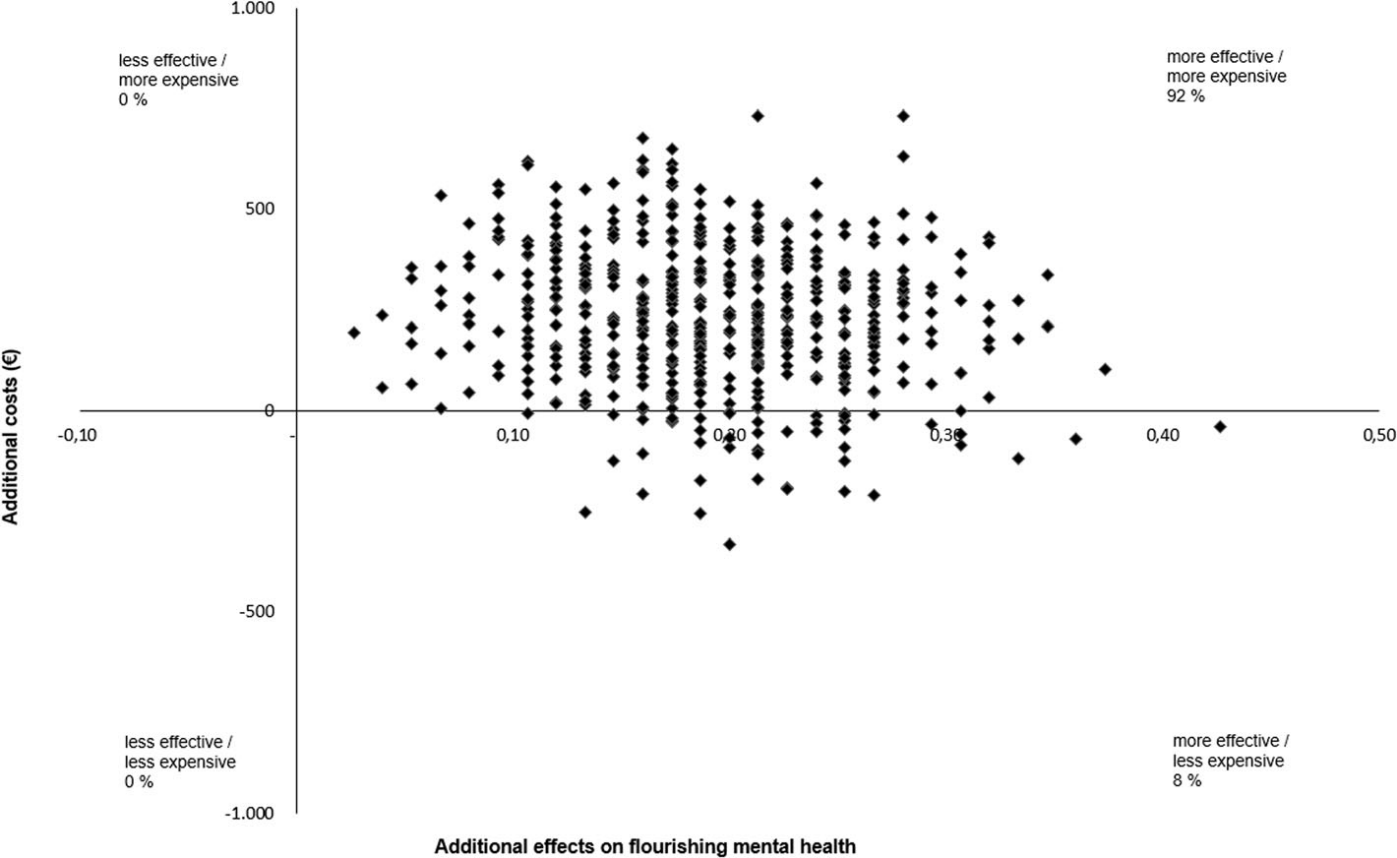
Cost-effectiveness plane of 2500 bootstrapped incremental cost-effectiveness ratios (ICERs) for flourishing, primary analysis

**Fig. 2:**
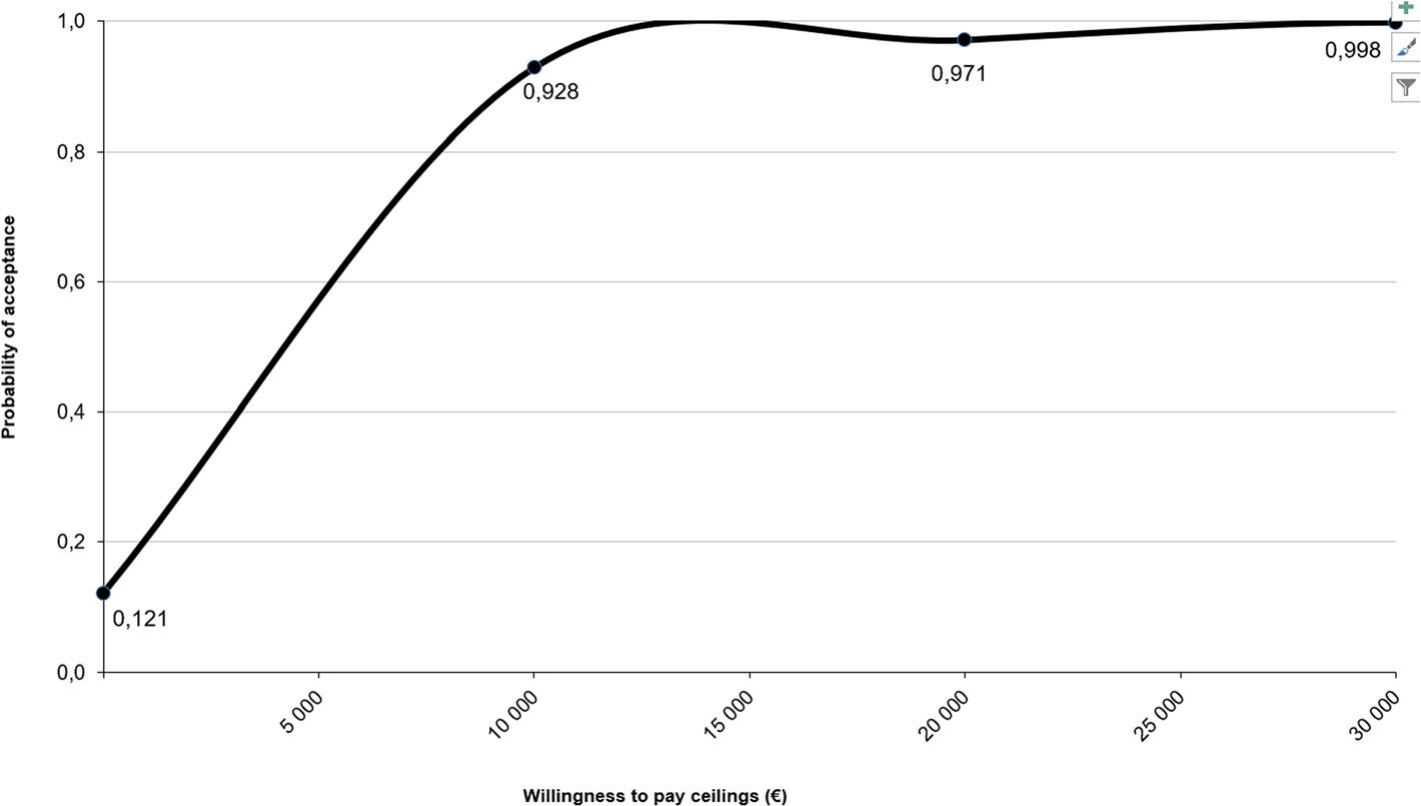
Cost-effectiveness acceptability curve: bootstrapped probability (*n* = 2500) that the guided PP-based intervention is acceptable compared to care as usual given varying willingness to pay ceilings, with flourishing as health outcome

The bootstrapped mean ICER shows that additional costs of €2359 ($2899) had to be paid to improve one person from low or moderate well-being to flourishing mental health. The bootstrapped median ICER showed additional costs of €1245 ($1530; Table 3). The majority of the plotted ICERs (92%) occurred in the northeast quadrant, indicating that the intervention produced health gains at additional costs (Fig. 1). All other plotted ICERs occurred in the southeast quadrant (8%), which implies health gains at lower costs. When there is no WTP, there is a 12% probability that the guided self-help intervention is more cost-effective relative to usual care (Fig. 2). This probability increases to 93% with a WTP ceiling for a favorable treatment outcome of €10,000 ($12,290). Conversely, with a probability of 80% the WTP is approximately €8000 ($9832).

**Table 3:** Cost-effectiveness analysis and sensitivity analysis with flourishing as health outcome

Flourishing	Total sample	Alternative scenarios		
		A	B	C
Costs, €^a^	239	196	617	223
Effect	0.19	0.19	0.19	0.20
ICER, €^b^	1245	1058	3240	1099
Distribution on the cost-effectiveness plane				
1st quadrant (northeast)	92	86	100	89
2nd quadrant (inferior: northwest)	0	0	0	0
3rd quadrant (southwest)	0	0	0	0
4th quadrant (superior: southeast)	8	14	0	11
WTP ceiling, %				
€ 0	12	18	4	14
€ 10,000	93	92	91	93
€ 20,000	97	97	96	97
€ 30,000	100	100	100	100

Scenario A = adjustment of the per participant intervention costs, based on actual costs for counseling (the number of extensive emails sent by each participant multiplied by €17); scenario B = intervention costs raised from €222 to €600; scenario C = completers only analysis (*n* = 112 intervention group and *n* = 125 control group)

^a^Costs per ‘disease-free’ year (i.e. one year in flourishing mental health) at 2014 prices

^b^Bootstrapped median, which is the 50th percentile of 2500 replications of the ICER

### Cost-effectiveness: Anxiety and depressive symptoms

The bootstrapped mean and median ICERs for treatment response on anxiety symptoms were €2959 ($3637) and €1095 ($1346) respectively, and for depressive symptoms €2578 ($3168) and €1189 ($1461; Table 4). The distribution of the 2500 bootstrapped ICERs on the cost-effectiveness plane as well as the WTP curve show similar results as has been found for flourishing: 91 and 90% of the ICERs were plotted in the northeast quadrant for anxiety and depressive symptoms respectively. The probability of accepting the intervention in favor of usual care with no WTP was 13% for both outcomes. At a WTP of €10,000, the probability of accepting the intervention had risen to 92 and 93% respectively.

**Table 4:** Cost-effectiveness analysis and sensitivity analysis with anxiety and depressive symptoms as health outcomes

	Total sample		Alternative scenarios					
			A		B		C	
	Anxiety	Depression	Anxiety	Depression	Anxiety	Depression	Anxiety	Depression
Costs, €^a^	239	239	196	196	617	617	223	223
Effect	0.22	0.21	0.22	0.21	0.22	0.21	0.26	0.23
ICER, €^b^	1095	1189	883	940	2785	2897	904	968
Distribution on the cost-effectiveness plane								
1st quadrant (northeast)	91	90	86	86	100	100	89	89
2nd quadrant (inferior: northwest)	0	0	0	0	0	0	0	0
3rd quadrant (southwest)	0	0	0	0	0	0	0	0
4th quadrant (dominant: southeast)	9	10	14	14	0	0	11	11
WTP ceiling, %								
€ 0	13	13	18	18	4	4	15	15
€ 10,000	92	93	93	93	91	90	92	92
€ 20,000	97	97	97	97	97	96	97	97
€ 30,000	100	100	100	100	100	100	100	100

Scenario A = adjustment of the per participant intervention costs, based on actual costs for counseling (the number of extensive emails sent by each participant multiplied by €17); scenario B = intervention costs raised from €222 to €600; scenario C = completers only analysis (*n* = 112 intervention group and *n* = 125 control group)

^a^Costs per ‘disease-free’ year (i.e. one year reliable improvement in depressive symptoms) at 2014 prices

^b^Bootstrapped median, which is the 50th percentile of 2500 replications of the ICER

### Sensitivity analyses

The results from the alternative scenarios show similar patterns for all three outcomes (see Table 3 for flourishing and Table 4 for anxiety and depression). Most mean and median bootstrapped ICERs were lowest in scenario A based on the actual intervention costs with mean ICERs between €1848 ($2271) and €2109 ($2592). Scenario B (intervention costs including the opportunity costs of the participants loss of leisure time) revealed the highest mean and median ICERs, while the completers-only analysis of scenario C was more comparable to scenario A. The percentage of ICERs on the cost-effectiveness planes for treatment response lies between 86% (scenario A) and 100% (scenario B). Furthermore, the probability of accepting the intervention over the control condition at no WTP lies between 4% (scenario B) and 18% (scenario A). In sum, these sensitivity analyses provide support for the robustness of the main analyses.

## Discussion

This health-economic evaluation is the first of its kind evaluating the cost-effectiveness of bibliotherapy based on a positive psychology framework. Participants with low or moderate levels of mental well-being received the book *This is Your Life* with email support or were placed on a wait-list (with full access to usual care). Results demonstrated that the intervention was effective at 6 months, showing significant improvements in mental well-being (from non-flourishing to flourishing mental health) while also decreasing both anxiety and depressive symptom severity [11]. However, owing to the intervention costs, the health care costs at 6 months were higher in the intervention group than the control group. The intervention costs were varied in sensitivity analyses, but all findings pointed in the same direction: substantial health gains can be expected from the intervention against an increase in health care costs of some €883~€4534 ($1085~$5572). From a decision-making point of view it might be worthwhile to note that the probability of regarding the intervention as cost-effective exceeds 90% at a willingness to pay of €10,000 ($12,290) per treatment responder.

These findings corroborate prior findings from cost-effectiveness analyses of (guided) bibliotherapy for depression [32, 33] and binge-eating disorder [34], although these interventions were based on cognitive behavioral therapy (CBT). Economic evaluations of web-based self-help interventions (mostly CBT-based) for mental health are more abundant [35]. To our knowledge, only one prior study evaluated the cost-effectiveness of a PP-based intervention or therapy [14]. This unguided web-based self-help intervention for people with mild to moderate depressive symptoms revealed substantially higher mean ICERs for mental well-being (€21,319) and depression (€9807) compared to the current study (€2359 and €2578 respectively). However, direct comparison of the ICERs is hindered by differences between both studies (e.g. outcome measures, societal vs. health care perspective, unguided web-based vs. guided bibliotherapy) despite some similarities in study design (e.g. recruitment strategy via newspapers in The Netherlands, mainly higher educated females and non-flourishers, a multicomponent PP structure, type of control condition). In addition, there are no known or generally accepted willingness-to-pay ceilings available for making a transition to a flourishing mental health state. Nonetheless, the cost-effectiveness acceptability curve indicates that a decision-maker has an 80% certainty that the intervention is deemed cost-effective at a WTP ceiling of roughly €8000 ($9832) and this likelihood increases to above 90% at the WTP ceiling of €10,000 ($12,290). Thus, the current study indicates that if there is a willingness to pay of at least €8000 for reaching a flourishing mental health state and avoiding anxiety and depression, guided PP-based bibliotherapy has a high likelihood to be seen as a cost-effective approach compared to usual care.

### Strengths and limitations

Main strengths of the study were its well-powered and randomized controlled design, the high adherence rates and results that appeared robust under sensitivity analyses. Also, as the self-selected sample of “well-being-seekers” recruited in the general Dutch population is congruent with usual recruitment strategies for self-help interventions in The Netherlands, the sample is representative for future applications of this intervention. However, we cannot generalize to the (unselected) general population because our sample overrepresented well-educated women in their late forties with paid jobs. An important limitation of the current study relates to the lack of an active control group wherein the self-help book could have been offered without email support, as was planned [20] but not feasible [11]. Prior studies demonstrated larger effects of guided CBT-based self-help than its unguided counterpart in improving mental well-being, anxiety and depression [13, 35, 36]. Hence, we cannot rule out the influence of adding email support to the PP-based bibliotherapy. Other limitations include the relatively short follow-up of 6 months and using this time-point for the extrapolation to an entire year (i.e. assuming a steady-state of the annualized costs and effects), the use of a wait-list control group (rather than usual care alone), unblinded participants, and the absence of assessing quality-adjusted life years (QALY’s) [27]. An advantage of using QALY’s is that a cost-utility analysis could have been added to the analyses to obtain the costs per QALY gained which is a *generic* health-related outcome that can be used across diseases and disorders. However, a strength of the current study was to use a *specific* health-related outcome that fitted well with the target group (people with languishing or moderate mental health) and the intervention aim (to establish the transition to flourishing mental health). In addition, the MHC-SF measuring mental well-being seems more sensitive to change [22] then the EQ-5D measuring QALY’s [37], although more research is needed to validate the theoretical cut-off scores for categorizing people into flourishing or not.

### Public health implications

A valuable approach for public mental health seems to enhance flourishing mental health because this status has been related to reduced risk of developing anxiety and depressive symptoms and mortality [8, 9, 38, 39]. The results of the present study demonstrated that an early intervention based on PP principles has the potential to promote a flourishing mental health status and substantially reduce anxiety and depressive symptom severity at some additional costs. The intervention could offer good value for money when there is a WTP of around €8000 ($9832), which corresponds to a probability of 80% that the intervention is more cost-effective compared to usual care. Even when the intervention costs are tripled by including the time costs of the participants, the WTP for a probability of 80% falls around €8000 ($9832). The economic costs of the intervention would rise from €2359 ($2899) for one additional person to improve from suboptimal well-being to flourishing in the main analysis to €4534 ($5572) when intervention costs are tripled. However, when there is no WTP for a treatment response, there is only a 12–13% probability that the intervention is more cost-effective relative to usual care. Thus, it remains a challenge to further optimize the cost-effectiveness of PP-based self-help interventions. Perhaps this can be achieved by shortening and refining the current program and focus on its most efficacious processes: enhancing positive relations, self-compassion and optimism [40]. In addition, it remains a great challenge to reach people with a lower socio-economic status, also with PP-based interventions.

Future research should replicate our findings and examine longer-term costs and benefits of PP interventions to promote flourishing. In this regard, we recommend large-scale implementation of the guided bibliotherapy program in public mental health or primary care wherein the intervention costs and effects are carefully monitored for more than one year. It is also of utmost importance to conduct economic evaluations of a guided versus unguided PP-based intervention which could shed light on the added value of therapist involvement. Furthermore, a cost-effectiveness study wherein a self-help intervention based on CBT is compared with a self-help intervention based on PP would be interesting because a prior study found that both CBT and PP where efficacious in ameliorating mental well-being and depression, but that the PP-based intervention was significantly more preferred, thus, having an impact on a larger population [41]. Overall, a PP-based self-help intervention with some guidance over the Internet has the potential to reach large groups of people via public mental health services, primary care and as additional service in mental health institutions. Also, a PP-based self-help intervention might hold promise as a worthwhile alternative to the predominant CBT-based self-help interventions. We hope the current study inspires researchers to plan and conduct economic evaluations alongside their trials to address this gap in the mental health promotion literature [13, 42].

## Conclusions

The results of this large randomized controlled trial demonstrated that a multicomponent positive psychology intervention was cost-effective on a reliable improvement in mental well-being as well as a reliable reduction in anxiety and depressive symptoms. The current study adds to prior knowledge because it is the first study worldwide demonstrating that mental health and flourishing can be substantially and cost-effectively improved via a positive psychology self-help intervention. This intervention is of great importance for public mental health and clinical practice because it has the potential to reach large groups of people through public mental health services and primary care with minimal investment.

## Abbreviations

CBT: Cognitive behavioral therapy
CHEERS: Consolidated Health Economic Evaluation Reporting Standards
EM: Expectation maximization
HADS: Hospital Anxiety and Depression Scale
ICER: Incremental cost-effectiveness ratio (ICER)
MCQ: Medical Consumption Questionnaire
MHC-SF: Mental Health Continuum-Short Form
PP: Positive Psychology
PPP: Purchasing power parity
QALY: Quality-adjusted life years
SPSS: Statistical Packages for Social Sciences
WTP: Willingness to pay
